# Plasma urea:creatinine ratio as a biomarker of gastrointestinal bleeding in dogs with anaemia

**DOI:** 10.1002/vms3.1286

**Published:** 2023-10-10

**Authors:** Ben Safrany, Andrea C. Holmes, Sophie Adamantos, Caroline Kisielewicz, Florence Juvet, Laura Macfarlane, Theresa McCann, Paula Valiente Diana, Fergus Allerton

**Affiliations:** ^1^ Department of Internal Medicine Paragon Veterinary Referrals (part of Linneaus Veterinary Limited) Wakefield UK; ^2^ Paragon Veterinary Referrals Wakefield UK; ^3^ Vet Oracle Telemedicine Clinical Director UK; ^4^ Department of Internal Medicine Southern Counties Veterinary Specialists Hampshire UK; ^5^ Department of Internal Medicine North Downs Specialist Referrals Bletchingley UK; ^6^ Department of Internal Medicine Davies Veterinary Specialists Hitchin UK; ^7^ Department of Internal Medicine Willows Referral Service Solihull UK

**Keywords:** anaemia, clinical pathology, dog, gastrointestinal

## Abstract

**Background:**

Gastrointestinal bleeding is a cause of anaemia in dogs. A reliable, non‐invasive biomarker to differentiate gastrointestinal bleeding from other causes of anaemia would be advantageous to direct clinical decisions in anaemic patients. Plasma urea:creatinine ratio is an accepted biomarker of upper gastrointestinal bleeding in human medicine.

**Objectives:**

The objective of this study was to evaluate plasma urea:creatinine ratio as a biomarker of gastrointestinal bleeding in a population of dogs with anaemia.

**Methods:**

This was a prospective cross‐sectional study of dogs with anaemia presenting to referral centres for the investigation of anaemia. Cases were categorised as having overt gastrointestinal bleeding (melena on presentation), occult gastrointestinal bleeding (historical and diagnostic findings consistent with gastrointestinal bleeding without melena at presentation) or anaemia of other cause (confident diagnosis other than gastrointestinal bleeding reached, normal diagnostic imaging of gastrointestinal tract). Urea:creatinine ratio at presentation was calculated by dividing urea (mg/dL) by creatinine (mg/dL).

**Results:**

Ninety‐five dogs were included. Plasma urea:creatinine ratio was not significantly different between dogs with overt or occult gastrointestinal bleeding or those with anaemia of other cause (median urea:creatinine ratio 25.8, 20.7 and 22.5, respectively). No significant difference in urea:creatinine ratio was found between dogs with upper and lower gastrointestinal bleeding (median urea:creatinine ratio 19.4 and 24.6, respectively).

**Conclusions:**

Plasma urea:creatinine ratio was not helpful in differentiating between dogs with anaemia resulting from gastrointestinal bleeding (overt or occult) and those with other causes of anaemia.

## Graphical abstract

Plasma urea:creatinine ratio was not significantly different between dogs with anaemia with overt or occult gastrointestinal bleeding or those with another cause of anaemia. No significant difference in plasma urea:creatinine ratio was found between dogs with upper and lower gastrointestinal bleeding.

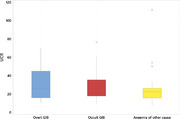

## 1 INTRODUCTION

Anaemia is the most common severe haematological abnormality encountered in dogs. Three pathological processes cause anaemia: haemorrhage, red blood cell destruction (haemolysis and haemophagocytosis) and decreased production (Stokol, 2017). In patients presenting for investigation of anaemia, it is desirable to identify the cause promptly to allow appropriate treatment to be initiated without extensive investigations. Although external bleeding can be quickly identified, internal haemorrhage can be more challenging to recognise and can cause profound blood loss (de Laforcade, 2017). In cases where gastrointestinal bleeding (GIB) is suspected, further investigation may include imaging and endoscopy to allow lesion visualisation and sampling (Tolbert, 2017). For these cases, lesion localisation to the upper or lower gastrointestinal tract is advantageous for planning procedures. Faecal occult blood testing is available; however, it can lack sensitivity, and due to the interference of dietary myoglobin with the assay, dietary modification for 3 days prior to performing the test is recommended (Cook et al., 1992; Villiers, 2021). An easily accessible, rapid test would be advantageous for clinical decision‐making.

In human medicine, the urea:creatinine ratio (UCR), calculated by dividing blood urea nitrogen (BUN) (mg/dL) by serum creatinine (mg/dL), is an accepted biomarker of upper gastrointestinal bleeding (Srygley et al., 2012). Cutoffs of 30 and 35 have been reported to have sensitivities of 51% and 20% and specificities of 91% and 90%, respectively (Srygley et al., 2012; Ziabari et al., 2019). The severity of an increase in urea may predict the severity of GIB, and urea is incorporated into the Blatchford clinical score, used to assign a risk level to patients with GIB and differentiate patients in need of urgent intervention from those that may be safely discharged (Blatchford et al., 2000).

Two previous veterinary studies, one retrospective and one part‐retrospective and part‐prospective, have demonstrated a significant difference in UCR between dogs with GIB and healthy control dogs (Prause & Grauer, 1998; Stiller et al., 2021). The first included only dogs with melena and/or haematemesis (overt GIB), and the second included both dogs with overt GIB and dogs where bleeding lesions were identified, without outward signs of bleeding (occult GIB). The latter study identified a significant difference in UCR between dogs with overt GIB and healthy controls but did not find a significant difference between dogs with occult GIB and healthy controls, or between dogs with bleeding localised to the upper or lower gastrointestinal tract. It is noteworthy that the controls in these studies were healthy dogs, and the effect of other diseases causing anaemia on UCR was not investigated. Before interpreting its value as a biomarker of GIB in a population of dogs with anaemia, it is important to first understand the effect of other causes of anaemia on UCR.

The primary objective of this study was to assess the suitability of UCR as a biomarker to identify overt and occult GIB in dogs with anaemia. The secondary objective was to evaluate the ability of the UCR to distinguish between upper and lower GIB. We hypothesised that in a population of dogs with anaemia, UCR would be significantly different between dogs with both overt and occult GIB and those with other causes of anaemia.

## 2 MATERIALS AND METHODS

### 2.1 Study design

This cross‐sectional study analysed client‐owned dogs presenting to eight referral centres in the United Kingdom (The Animal Health Trust, Davies Veterinary Specialists, Dick White Referrals, Langford Vets Small Animal Referral Hospital, North Downs Specialist Referrals, Pride Veterinary Centre, Southern Counties Veterinary Specialists and Willows Referral Service) between November 2017 and July 2018 for the evaluation of anaemia. Dogs were prospectively enrolled and eligible for inclusion if they were anaemic at presentation (packed cell volume [PCV]/haematocrit [HCT] <35%/0.35) and if BUN or urea and serum creatinine were measured at the time of presentation. Cases were excluded if BUN or urea and serum creatinine were not measured, where it could not be confirmed that dogs were fasted for a minimum of 8 h before venepuncture or where complete medical records were not available to review.

### 2.2 Data extraction

Data collected included signalment (age, breed, sex and neuter status), historic information (drugs administered in the previous 4 weeks, presence or absence of melena, haematemesis, epistaxis or haemoptysis in the previous 72 h), clinical variables on presentation (body weight, body condition score [BCS], heart rate, respiratory rate, temperature, rectal examination, estimated percentage of dehydration and systolic blood pressure [SBP]), clinicopathological parameters on presentation (PCV or HCT, serum creatinine, urea and urine specific gravity), results of diagnostic imaging (ultrasound, radiographs, computed tomography and endoscopy) and final diagnosis (as assessed by the primary case clinician). Clinical chemistry parameters were measured by a variety of in‐house and external laboratory analysers. Urea in mmol/L was converted to BUN in mg/dL (by dividing urea in mmol/L by 0.357). Creatinine in μmol/L was converted to mg/dL (by dividing creatinine in μmol/L by 88.42) to allow the calculation of the ratio between BUN and creatinine. PCV was used preferentially over HCT; however, if only HCT was recorded, it was converted to an estimated PCV (by multiplying HCT by 100) to facilitate data analysis. Upper gastrointestinal bleeding was defined as bleeding proximal to the duodenal‐jejunal junction. Where possible, the pathological process considered to be the cause of anaemia (as agreed by the primary authors) was recorded. If a clear cause was not found, or there were multiple possible processes, cases were excluded from the analysis of pathological processes.

### 2.3 Definitions of gastrointestinal bleeding

Dogs were categorised as having ‘overt’, ‘occult’, ‘unlikely’ GIB or anaemia of other cause. Dogs categorized with overt GIB had melena on physical examination by a specialist or resident in internal medicine at the time of presentation. Dogs categorised as having anaemia of other cause had a firm alternative diagnosis and cause of anaemia identified. It was a requirement of this group to have abdominal imaging (ultrasound or computerized tomography (CT) scan) performed. Dogs categorised as occult GIB had historical (history of haematemesis and/or melena reported in the 72 h prior to presentation) and/or diagnostic investigation findings (ultrasound, CT, endoscopy or post‐mortem) consistent with gastrointestinal haemorrhage (such as recent haematemesis, melena or a gastrointestinal mass) and were considered to have significant GIB by the primary clinician; however, they lacked melena on physical examination at the time of blood sampling and investigations. Dogs categorised as unlikely GIB had no clinical signs or diagnostic investigation findings consistent with gastrointestinal haemorrhage; however, a confident alternative diagnosis was not made, or abdominal imaging was not performed.

### 2.4 Data analysis

Analysis was performed on data from dogs with overt and occult GIB and anaemia of other cause. Dogs where GIB was considered unlikely but not entirely excluded were not included in statistical analysis.

Demographic and baseline characteristics, laboratory findings and UCR for the three groups undergoing analysis were assessed for normality with Shapiro–Wilk tests. Since some failed this test, Kruskal–Wallis tests were used throughout to compare continuous variables. Any tests that generated significant results were further investigated using Dunn multiple comparisons with Benjamini–Hochberg adjustment. Categorical variables were compared using Fisher's exact tests; any variables generated significant results were further investigated using post hoc multiple comparisons with Benjamini–Hochberg adjustment. For selected variables, a comparison was also made between the combined overt and occult groups and the anaemia of the other cause group. An association between continuous variables was tested by Spearman rank correlations. Any tests achieving *p* < 0.05 were considered statistically significant. All analyses were performed using R version 3.6.2 or Minitab 19.

Estimating standard deviation from the ranges presented by Stiller et al. (2021) and assuming an effect size of 10, a power of 0.80 and a significance level of 0.05, a sample size calculation suggested 24 dogs per group.

## 3 RESULTS

### 3.1 Study population

In total, 110 dogs were eligible for inclusion. Of these, 28 were classified as having overt GIB, 27 occult GIB and 40 anaemia of other cause, meaning 95 cases were used for statistical analysis. GIB was considered unlikely in the remaining 15 dogs.

The signalment of dogs with overt and occult GIB and anaemia of other cause are described in Table 1. In total, 36 breeds were represented along with 10 crossbreeds. No significant differences in signalment or breed distribution between the groups were found.

**TABLE 1:** Signalment of a population of dogs with anaemia, grouped by those with overt gastrointestinal bleeding, occult gastrointestinal bleeding and those with anaemia of other cause.

	Overt GIB	Occult GIB	Anaemia of other cause	*p*
*n*	28	27	40	‐
Age in months, median (range)	84 (11–176)	108 (4.5–154)	83 (6–156)	0.52^a^
Female/male	10/18 (36%/64%)	13/14 (48%/52%)	15/25 (38%/62%)	0.58^b^
Neutered/entire	21/7 (75%/25%)	23/4 (85%/15%)	30/10 (75%/25%)	0.61^b^
Bodyweight (kg), median (range)	25.0 (7.0–47.0)	17.1 (4.1–38.0)	16.1 (5.5–39.7)	0.20^a^
Labrador *n* (%)	8 (28.6)	4 (14.8)	6 (15.0)	0.30^b^
Miniature Schnauzer *n* (%)	1 (3.6)	1 (3.6)	7 (17.5)	0.14^b^
Springer Spaniel *n* (%)	3 (10.7)	1 (3.7)	4 (10.0)	0.72^b^
Cocker Spaniel *n* (%)	1 (3.6)	4 (14.8)	2 (5.0)	0.32^b^

*Note*: Medians (ranges) are presented for continuous data and number (%) for categorical data.

Abbreviation: GIB, gastrointestinal bleeding.

^a^ Kruskal–Wallis test.

^b^ Fisher's exact test.

The historical information at the time of presentation is summarised in Table 2. Owners of dogs with GIB (overt and occult combined) were more likely to report noticing melena or haematemesis than owners of dogs with anaemia of other cause (*p* < 0.001 and *p* = 0.009, respectively). Dogs with occult GIB were more likely to have recently been prescribed medication (*p* = 0.008), particularly glucocorticoids (*p* = 0.020) when compared with dogs where anaemia of other cause.

**TABLE 2:** Historical findings of a population of dogs with anaemia, grouped by those with overt gastrointestinal bleeding, occult gastrointestinal bleeding and those with anaemia of other cause.

	Overt GIB	Occult GIB	Anaemia of other cause	*p* ^a^
*n*	28	27	40	–
Melena	26 (92.9%)a	14 (51.9%)b	0c	<0.001
Haematemesis	5 (17.9%)a	4 (14.8%)a	0c	0.009
Epistaxis	2 (7.1%)	0	0	0.16
Medication administration within 28 days of presentation	19 (67.9%)ab	24 (88.9%)a	20 (50.0%)b	0.003
Glucocorticoids	9 (32.1%)ab	12 (44.4%)a	6 (15.0%)b	0.023
NSAIDs	3 (10.7%)	6 (22.2%)	6 (15.0%)	0.49
Antimicrobials	7 (25.0%)	9 (33.3%)	8 (20.0%)	0.45
Gastroprotectants	8 (28.6%)	5 (18.5%)	3 (7.5%)	0.07
Antithrombotics	0	1 (3.7%)	0	‐

*Note*: Data are presented as number (%). Where significant, post hoc tests were undertaken, and significant differences are identified between the groups that do not share a common letter.

Abbreviations: GIB, gastrointestinal bleeding; NSAID, non‐steroidal anti‐inflammatory drug.

^a^ Fisher's exact test.

Clinical examination findings are summarised in Table 3. Systolic blood pressure was recorded in 22/28 (79%) dogs with overt GIB, 23/27 (85%) dogs with occult GIB and 33/40 (82%) dogs with anaemia of other cause. It was lower in dogs with occult GIB compared with dogs with overt GIB and anaemia of other cause (*p* = 0.043 and *p* = 0.039, respectively, median SBP 124, 140 and 141 mmHg), but not significantly different between the latter two groups (*p* = 0.91). Body condition score was lower in the occult GIB group than the overt GIB or anaemia of other cause groups (both *p* < 0.001, median BCS 3, 5 and 4.5, respectively), but not significantly different between the latter two groups (*p* = 0.44).

**TABLE 3:** Clinical examination findings (at initial examination) of a population of dogs with anaemia, grouped by those with overt gastrointestinal bleeding, occult gastrointestinal bleeding and those with anaemia of other cause.

	Overt GIB	Occult GIB	Anaemia of other cause	*p* ^a^
Heart rate (bpm)	120 (80–160)	126 (76–200)	120 (68–200)	0.47
Respiratory rate (bpm)	24 (16–60)	32 (16–60)	34 (20–60)	0.25
Temperature (°C)	38.4 (37.1–40.0)	38.65 (37.5–39.8)	38.5 (36.1–39.5)	0.22
Systolic blood pressure (mmHg)	140 (109–215)a	124 (98–200)b	141 (92–230)a	0.027
BCS (1–9)	5 (3–9)a	3 (1–6)b	4.5 (2–8)a	<0.001

*Note*: Values are expressed as median (range). Where significant, post hoc tests were undertaken, and significant differences are identified between the groups that do not share a common letter.

Abbreviations: BCS, body condition score; bpm, beats/breaths per minute; GIB, gastrointestinal bleeding.

^a^ Kruskal–Wallis test.

### 3.2 Clinicopathological findings and UCR

**FIGURE 1:**
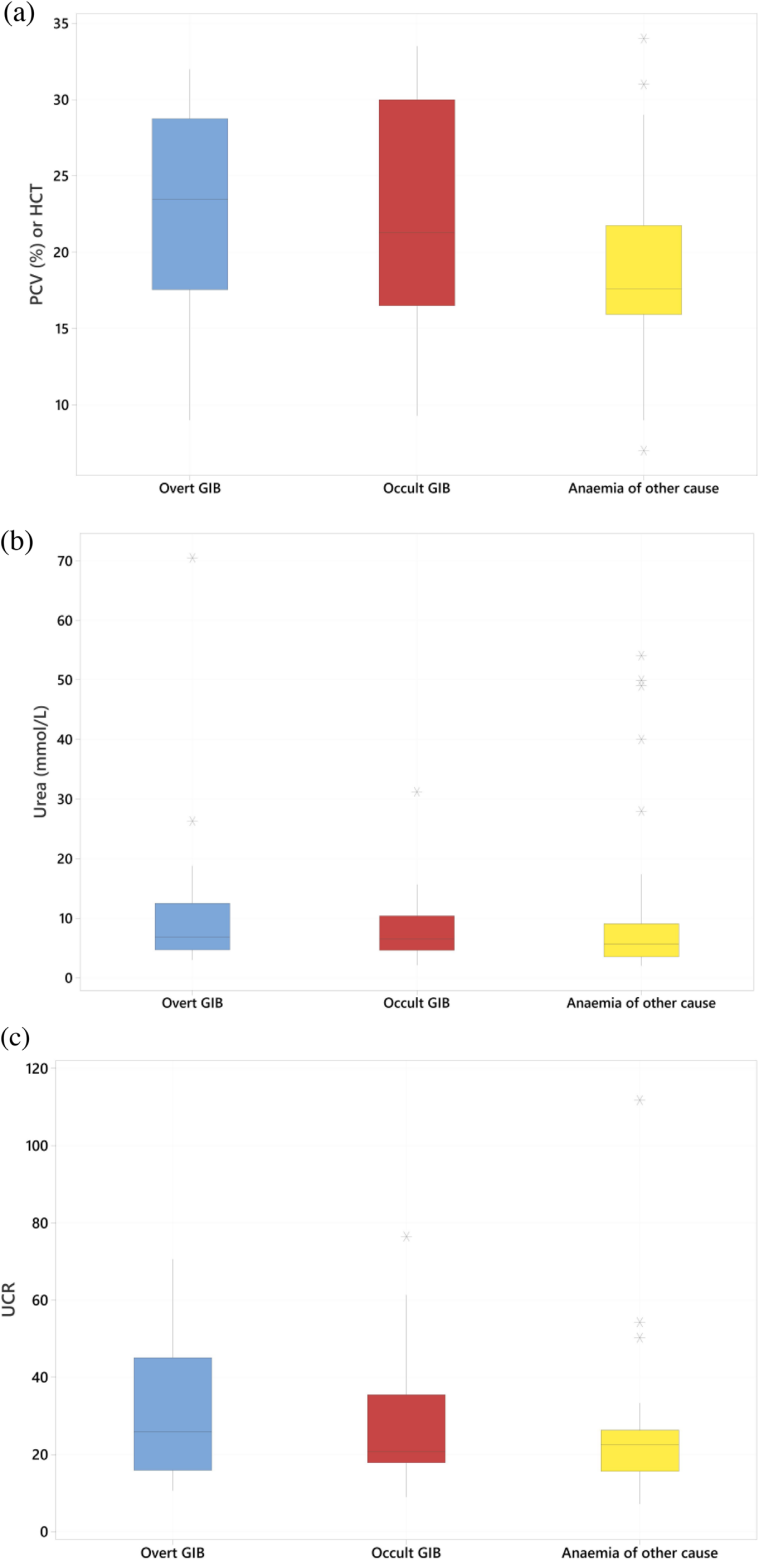
Box and whisker plots comparing (a) packed cell volume (PCV), (b) urea and (c) urea:creatinine ratio (UCR) in dogs with anaemia with overt gastrointestinal bleeding (GIB), occult gastrointestinal bleeding and with anaemia of other cause. HCT, haematocrit.

Clinicopathological findings are summarised in Table 4 and Figure 1a–c. No significant difference in plasma urea (median overt GIB 6.9 mmol/L, occult GIB 6.5 mmol/L, anaemia of other cause 5.6 mmol/L; *p* = 0.50) or UCR (median overt 25.8, occult 20.7, anaemia of other cause 22.5; *p* = 0.34) was found between the groups. PCV was significantly lower in the anaemia of the other cause group (median 17.6%) than in the two GIB groups (median overt 23.5% *p* = 0.039, occult 21.3% *p* = 0.042). There was no significant difference between the GIB groups (*p* = 0.81).

**TABLE 4:** Clinicopathological findings (at initial presentation) of a population of dogs with anaemia, grouped by those with overt gastrointestinal bleeding, occult gastrointestinal bleeding and those with anaemia of other cause.

	Overt GIB	Occult GIB	Anaemia of other cause	*p* ^a^
PCV (%)	23.5 (9–32)a	21.3 (9–33)a	17.6 (7–34)b	0.020
Urea (mmol/L)	6.9 (3.0–70.5)	6.5 (2.1–31.2)	5.6 (2.0–54.1)	0.50
Creatinine (μmol/L) median (range)	72 (29–290)	75 (21–304)	68 (26–791)	0.83
UCR median (range)	25.8 (10.6–70.6)	20.7 (9.0–76.5)	22.5 (7.2–111.8)	0.34

*Note*: Values are expressed as median (range). Where significant, post hoc tests were undertaken, and significant differences are identified between the groups that do not share a common letter.

Abbreviations: GIB, gastrointestinal bleeding; PCV, packed cell volume; UCR, urea:creatinine ratio.

^a^ Kruskal–Wallis test.

When dogs recently treated with glucocorticoids were compared with those that had not recently been treated with glucocorticoids, UCR was not significantly different (median 22.1 and 22.5, respectively; *p* = 0.06). UCR was not significantly different in dogs recently treated with gastroprotectants compared with those that were not (median 21.9 and 22.4, respectively; *p* = 0.53).

When assessed across all groups, no significant correlation was found between UCR and age, BCS, heart rate or systolic blood pressure (Table 5). A significant negative correlation was demonstrated between UCR and bodyweight (*p* < 0.001) and PCV (*p* = 0.001). Of these variables with a significant correlation with UCR, when urea and creatinine were assessed individually, the only significant (positive) correlation was found between creatinine and bodyweight (*r_s_* = 0.350, *p* = 0.001).

**TABLE 5:** Spearman rank correlations between urea:creatinine ratio and other variables in a population of dogs with anaemia.

Variable	Correlation	95% Confidence interval	*p*
Age (months)	0.03	(−0.18, 0.23)	0.80
Body weight (kg)	−0.39	(−0.55, −0.20)	<0.001
BCS (0–9)	−0.14	(−0.34, 0.07)	0.18
Heart rate (bpm)	0.04	(−0.17, 0.24)	0.74
Systolic blood pressure (mmHg)	0.16	(−0.07, 0.37)	0.17
PCV (%)	−0.34	(−0.51, −0.14)	0.001

Abbreviations: BCS, body condition score; PCV, packed cell volume; bpm, beats/breaths per minute.

The final diagnosis reached for dogs presenting with overt or occult GIB is summarised in Table 6. In 4/28 dogs with overt GIB and 1/27 dogs with occult GIB, the cause of bleeding was not found; due to the spontaneous resolution of bleeding before, the investigation was completed in four dogs and an owner‐directed decision to euthanise in one dog. No significant differences were found between occult or overt bleeding in the frequencies of underlying diseases. In the anaemia of the other cause group, a final diagnosis of IMHA was reached in 20/40 dogs, five of which had non‐regenerative IMHA. Note that 7/40 dogs had a final diagnosis of haemoabdomen; in six cases, this was secondary to intraabdominal neoplasia; in one case, the source of abdominal bleeding was not reached, and the dog was euthanised before a firm diagnosis was reached. Three dogs were anaemic due to non‐gastrointestinal neoplasia, including a cardiac mass, a splenic mass causing microangiopathic anaemia and a hepatic sarcoma. Two dogs had neoplasia involving the bone marrow. Two dogs had chronic kidney disease. Individual dogs were diagnosed with acute kidney injury, babesiosis, hepatic failure, immune‐mediated polyarthritis, myelofibrosis and pyometra.

**TABLE 6:** Final diagnosis reached in a population of dogs with anaemia with gastrointestinal bleeding.

Diagnosis	Overt GIB (*n* = 28) *n* (%)	Occult GIB (*n* = 27) *n* (%)	*p* ^a^
Chronic kidney disease	0	1 (3.7)	0.49
Foreign body	1 (3.6)	0	1.00
Gastrointestinal neoplasia	3 (10.7)	7 (25.9)	0.18
Hypoadrenocorticism	0	1 (3.7)	0.49
Inflammatory gastrointestinal disease	5 (17.9)	7 (25.9)	0.53
Immune‐mediated thrombocytopenia	5 (17.9)	5 (18.5)	1.00
Medication related	8 (28.6)	3 (11.1)	0.18
No cause found	4 (14.3)	1 (3.7)	0.35
Portal hypertension	0	2 (7.4)	0.24
Toxicity (rodenticide toxicity)	2 (7.1)	0	0.49

Abbreviation: GIB, gastrointestinal bleeding.

^a^ Fisher's exact test.

**FIGURE 2:**
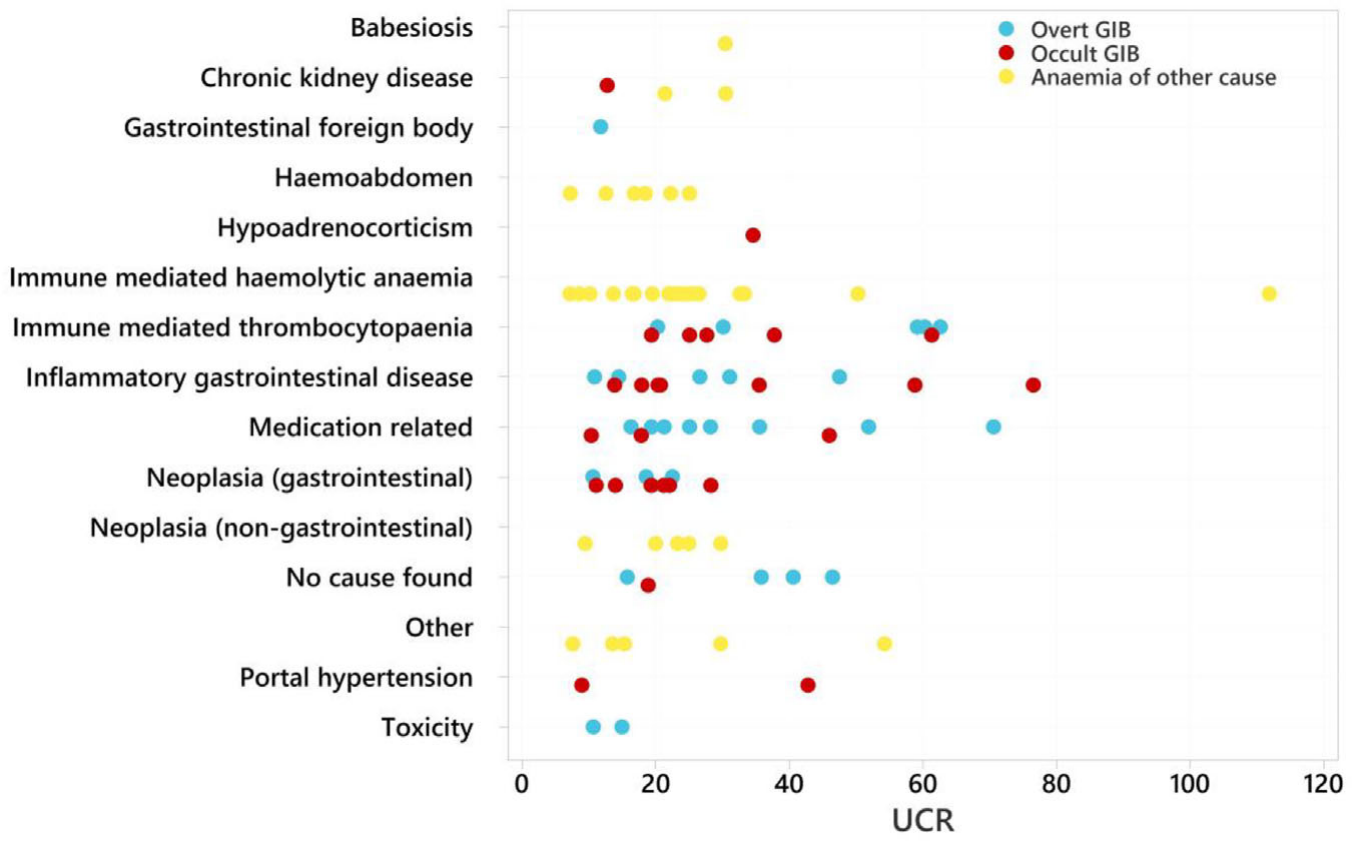
Scatter plot demonstrating urea creatinine ratio (UCR) associated with different causes of gastrointestinal bleeding (GIB) in a population of dogs with anaemia.

When UCR was compared between diagnoses, a significant difference was found (*p* = 0.043), with the highest UCR observed in immune‐mediated thrombocytopenia (ITP) and the lowest in patients with haemoabdomen and gastrointestinal neoplasia (median 33.9, 18.5 and 19.3, respectively) (Table 7, Figure 2). No significant difference between dogs grouped by final diagnosis was found in creatinine. BCS differed between final diagnosis (*p* = 0.038) with dogs with neoplasia (gastrointestinal or non‐gastrointestinal) having the lowest BCS, and dogs with IMHA having the highest BCS (median 3.5, 3.0 and 6.0, respectively).

**TABLE 7:** Final diagnosis and pathophysiology of bleeding, packed cell volume and urea:creatinine ratio (UCR) in a population of dogs with anaemia.

Diagnosis	*n*	PCV (%) Median (range)	UCR Median (range)
Haemoabdomen	7	18.0 (17.0–31.0)	18.5 (7.3–25.1)
Immune‐mediated haemolytic anaemia	20	16.3 (7.0–23.0)	22.9 (7.2–111.8)
Inflammatory gastrointestinal disease	12	24.4 (11.0–33.0)	23.7 (10.9–76.5)
Immune‐mediated thrombocytopenia	10	16.2 (9.0–25.7)	33.9 (19.4–62.6)
Medication related	11	24.0 (9.3–31.0)	25.1 (10.4–70.6)
Neoplasia (gastrointestinal)	10	23.1 (16.5–33.5)	19.3 (10.6–28.3)
Neoplasia (non‐gastrointestinal)	5	19.8 (9.4–25.8)	23.3 (9.5–29.8)
*p*	–	0.016^a^	0.043^a^

Abbreviations: GIB, gastrointestinal bleeding; PCV, packed cell volume.

^a^ Kruskal–Wallis test.

Based on the pathological process causing anaemia, anaemia was categorised as due to blood loss, decreased erythrocyte production or haemolysis in 59/89 (66%), 12/89 (13%) and 18/89 (20%) dogs, respectively. In the remaining six dogs, either a clear cause was not found or multiple processes could have contributed to the anaemia. Median UCR was 22.1 in cases where blood loss was the cause of anaemia, 20.7 in cases with reduced production of erythrocytes and 22.9 in cases with haemolysis. A significant difference between processes was not observed (*p* = 0.50).

A bleeding lesion was confidently demonstrated in 23/55 (42%) dogs with gastrointestinal bleeding (11/28 [39%] overt, 12/27 [44%] occult; based on ultrasound in 16/23, combined ultrasound and CT in 3/23, flexible endoscopy in 3/23 and CT alone in 1/23). The primary lesion was detected in the stomach in 14/23 (61%) cases, the duodenum in 3/23 (13%) cases, the jejunum in 4/23 (17%) cases and the colon in 2/23 (9%) cases. There was no significant difference in UCR between dogs with lesions in the upper gastrointestinal tract (stomach and duodenum) and the lower gastrointestinal tract (jejunum, ileum and colon) (median UCR 19.4 and 24.6, respectively, *p* = 0.67).

## 4 DISCUSSION

In our study population of dogs with anaemia, UCR was not significantly different in dogs with overt or occult GIB or anaemia of other cause. This is in contrast to previous studies, which identified a difference between dogs with overt GIB and control dogs. Our finding of a median UCR of 25.8 in dogs with overt GIB is consistent with these studies, which reported median UCRs ranging from 19.4 to 34 (Prause & Grauer, 1998; Waldrop et al., 2003; Stiller et al. 2021; Table 8). However, the median UCR of 22.4 in dogs with anaemia of other cause in the present study is higher than the previously described median UCRs of between 14.4 and 16.2 in healthy control dogs (Table 8). Control dogs in previous studies included healthy dogs presenting for blood donation, elective procedures or as participants in other studies. It is possible that pathology causing anaemia other than GIB may lead to an elevated UCR. This may further diminish the diagnostic utility of UCR for detecting GIB in a clinical situation where it is desirable to differentiate patients with GIB from those with other systemic illnesses.

**TABLE 8:** Summary of median urea:creatinine ratio in dogs with overt gastrointestinal bleeding and control populations reported in the literature.

	Overt GIB	Control
Prause and Grauer (1998)	27.6	14.4 (Healthy)
Waldrop et al. (2003)	34	–
Lobetti (2012)	–	15.36 (Healthy)
Stiller et al. (2021)	19.4	16.2 (Healthy)
Current work	25.8	22.4 (Anaemic)

Abbreviation: GIB, gastrointestinal bleeding.

It has been proposed that GIB causes an elevation in serum urea through the gastrointestinal absorption of nitrogenous compounds produced following the digestion of whole blood. Studies have shown that the oral administration of citrated blood to humans leads to an elevation in serum urea of 25%–35% (Schiff et al., 1942). GIB may also increase UCR by causing a prerenal azotaemia, during which there is renal tubular reabsorption of urea, but not creatinine. Evidence to support this theory was provided by a study where dogs were repeatedly bled and developed UCR elevation (Atata et al., 2019).

An increase in UCR was not always observed in dogs with overt GIB. This could be due to the peak in serum urea being missed at the time of sampling. It has been shown that serum urea peaks between 4.5 and 10 h following the administration of blood to dogs (Yuile & Hawkins, 1941) and in humans returns to baseline by 48 h (Schiff et al., 1942). The timing of the effects of pre‐renal azotaemia on serum urea concentrations has not been investigated. It would be expected that the effect would develop quickly following bleeding and return to normal once the initial hypovolaemia had been corrected. In an attempt to identify patients with the highest UCRs, only dogs with melena at the time of blood sampling were included in the overt GIB group. In humans orally administered blood on a single occasion, melena can persist for up to 5 days (Schiff et al., 1942). If the same is true in dogs, melena at the time of sampling may reflect GIB several days earlier, and the peak in serum urea may be missed. If this is the case, the usefulness of UCR as a biomarker would be diminished. In our study population, the highest UCR in dogs with GIB was seen in dogs diagnosed with ITP. In ITP, steady, consistent mucosal bleeding is expected, delivering a steady supply of blood into the gastrointestinal tract. The lowest UCR was seen in dogs with gastrointestinal neoplasia, where bleeding may be more intermittent.

Dogs with anaemia of other cause had higher UCRs than previously reported in healthy control dogs. In 2012, Lobetti (2012) reported a median UCR of 41.35 (range: 17.27–94.86) in dogs with babesiosis, demonstrating that significant increases in UCR can be observed in pathology other than GIB. Significant increases in UCR in dogs with experimentally induced haemoglobinaemia, anaemia and haemoglobinaemia, or naturally occurring IMHA were not detected. The author concluded that the increases in UCR were unlikely to be related to renal disease, increased haemoglobin load on the liver, haemoglobin interference or gastrointestinal bleeding and proposed that further investigation be performed to understand the mechanism causing elevated UCR.

In humans, an increased UCR has been demonstrated in patients with heart failure and receiving dialysis (Inaguma et al., 2018; Sood et al., 2015) and is associated with a poorer prognosis. It has been hypothesised that increased urea may result from volume depletion or neurohormonal activation. This could explain the increase in UCR seen in dogs with significant blood loss; however, the increase in UCR observed in haemoabdomen patients, where blood loss is often significant, was relatively modest compared to other disease processes. In the dogs with anaemia of other cause, the largest increases in UCR were seen in dogs with IMHA and non‐gastrointestinal neoplasia. Patients with IMHA are expected to be normovolaemic, and the mechanism leading to an increased UCR is not clear. It has been demonstrated that in dogs with cancer, 35% have mild to severe muscle wasting, which could lead to a reduction in serum creatinine leading to an increase in UCR. In our population, dogs with neoplasia had lower BCS than other groups; however, a difference in creatinine was not observed.

The finding that dogs with occult GIB were more likely to have received medication in the 4 weeks prior to presentation could either reflect the incidence of medication‐related GIB, or referring veterinary surgeons administering treatment trials with gastroprotectants prior to referral. This may be more likely in dogs with a milder, potentially more chronic occult presentation. It did not appear that medication‐related GIB was more associated with an overt or occult presentation of GIB. Dogs with occult GIB were more likely to have been treated with glucocorticoids. It has previously been reported that glucocorticoid use is associated with a higher UCR (Stiller et al., 2021); however, a significant increase in UCR in glucocortoicoid‐treated dogs was not observed in our population. In the present study, information on glucocorticoid dose was not collected, meaning it is not possible to compare glucocorticoid dosages between studies.

Dogs with occult GIB had a lower systolic blood pressure than those with overt GIB and anaemia of other cause. The median systolic blood pressure of ∼120 mmHg in the occult group is considered normal, whereas the median blood pressure of 140/141 mmHg reported in the overt/anaemia of other cause groups is marginally elevated (Acierno et al., 2018). This may reflect a less acute disease process in dogs with occult GIB and increased sympathetic nervous system activation in the overt GIB and anaemia of other cause groups. Dogs with occult GIB also had a lower body condition score, possibly reflecting a longer disease process allowing reduced body mass to be noted. However, an association between the final diagnosis and the detection of overt or occult GIB was not found.

Dogs with anaemia of other cause were found to have a lower PCV than those with GIB, and those with the lowest PCV were diagnosed with IMHA and ITP. IMHA is typically associated with a markedly low PCV at presentation (Mason et al., 2003; McAlees, 2010; Scott‐Moncrieff, 2001; Piek et al., 2008; Piek, 2011). Although anaemia is commonly reported in ITP (Botsch et al., 2009), it is usually described as being milder than our study population (Putsche & Kohn, 2008).

A negative association between UCR and body size was identified and has previously been reported, and this is thought to be a consequence of higher muscle mass and therefore creatinine in larger, more muscled breeds (Braun et al., 2003; Palm, 2017). A negative correlation between UCR and PCV was also demonstrated. This may be related to increased disease severity (and therefore lower circulating blood volume and increased neurohormonal activation) with lower PCV.

The negative correlation between PCV and UCR in combination with the significantly lower PCV in the non‐GIB group may account for, or have contributed to the absence of a significant difference between groups. However, the study population is representative of dogs presenting for anaemia investigation in the United Kingdom.

We were unable to demonstrate a difference in UCR between upper and lower intestinal bleeding, the same finding was reported by Stiller et al. (2021). This finding may reflect physiological differences in the sites of amino acid absorption between species. In their study, Stiller et al. (2021) proposed that colonic haemorrhage could result in the increased hepatic production of urea as a consequence of the absorption of protein breakdown products and/or ammonia. We considered the junction of the upper and lower gastrointestinal tract to be the distal duodenum. In humans, the upper and lower gastrointestinal tract is divided by the ligament of Treitz, an eponym for the suspensory ligament of the duodenum which indicated the junction between the duodenum and the jejunum (Tomizawa et al., 2015). The ligament of Treitz does not exist in dogs due to anatomical differences (Watkins et al., 1993); however, this classification has been used in previous veterinary studies (Stiller et al., 2021). In six dogs, a bleeding lesion was identified aboral to the distal duodenum (the jejunum in four dogs and the colon in two dogs). Three were in the overt GIB group and presented with melena. Lower GIB is more typically associated with haematochezia; however, it has been suggested that in cases with prolonged intestinal transit time that lesions in the caecum and colon can be associated with melena (Tefft, 2017). In human patients, bleeding in the colon has been reported to produce melenic stool (Aristodemou et al., 1992; Vipond et al., 1990). It is also possible that these patients had additional sites of bleeding higher in their gastrointestinal tract causing melena.

The present study has several limitations. It was not possible to comprehensively exclude occult GIB in the anaemia of the other cause group, and it was only possible to ascertain that their anaemia was not a consequence of GIB. A large proportion of dogs were receiving medication at the time of enrolment, which may have impacted results. Many of these dogs were receiving glucocorticoids and/or NSAIDs prior to enrolment. Previous studies (Stiller et al., 2021) have excluded cases of glucocorticoid medication due to the association with increases in UCR. We chose not to exclude patients pre‐treated with glucocorticoids as a significant difference in UCR was not found in treated patients. It is possible that this finding reflected a type‐2 error as a consequence of sample sizes not being calculated on the basis of this comparison. GIB was excluded clinically and based on diagnostic imaging; however, subclinical GIB could not be entirely ruled out in the anaemia of the other cause group. A wide variety of in‐house and external laboratory analysers were used for the measurement of urea and creatinine. The effect of this may be mitigated by the values being incorporated into a ratio, and for UCR to be a helpful biomarker, it would need to be utilisable on in‐house analysers to aid decision‐making. Only a small proportion of dogs had actively bleeding lesions demonstrated with endoscopy, meaning that comparisons between upper and lower GIB were likely underpowered. The sample size for the primary outcome measure was reached; however, subsequent analysis was likely to be underpowered. The cohort of cases was enrolled at referral institutions and therefore may not be representative of a more general population of dogs presenting for anaemia investigation.

In conclusion, in our study population, UCR had a poor ability to discriminate between dogs with anaemia with overt or occult GIB, or where anaemia was due to other cause and could not discriminate between upper or lower GIB. On this basis, it seems to have poor utility to establish the cause of anaemia in dogs presenting for anaemia investigation. It seems likely that other disease processes can also affect UCR, and further investigation would be needed to understand the pathophysiological process behind this.

## AUTHOR CONTRIBUTIONS

Ben Safrany: Data curation; formal analysis; investigation; writing—original draft; writing—review and editing. Sophie Adamantos: Investigation; writing—review and editing. Caroline Kisielewicz: Investigation; writing—review and editing. Florence Juvet: Investigation; writing—review and editing. Laura Macfarlane: Investigation; writing—review and editing. Theresa McCann: Investigation; writing—review and editing. Paula Valiente: Investigation; writing—review and editing. Fergus Allerton: Investigation; writing—review and editing.

## CONFLICT OF INTEREST STATEMENT

The authors declare no conflicts of interest.

## ETHICS STATEMENT

The study was approved by the Animal Health Trust ethical review panel (reference 34–2017).

### PEER REVIEW

The peer review history for this article is available at https://publons.com/publon/10.1002/vms3.1286.

## Data Availability

The data that support the findings of this study are available from the corresponding author upon reasonable request.
